# Manipulation of Dirac Cones in Mechanical Graphene

**DOI:** 10.1038/srep18107

**Published:** 2015-12-15

**Authors:** Toshikaze Kariyado, Yasuhiro Hatsugai

**Affiliations:** 1 Division of Physics, Faculty of Pure and Applied Sciences, University of Tsukuba, Tsukuba, Ibaraki 305-8571, Japan

## Abstract

Recently, quantum Hall state analogs in classical mechanics attract much attention from topological points of view. Topology is not only for mathematicians but also quite useful in a quantum world. Further it even governs the Newton’s law of motion. One of the advantages of classical systems over solid state materials is its clear controllability. Here we investigate mechanical graphene, which is a spring-mass model with the honeycomb structure as a typical mechanical model with nontrivial topological phenomena. The vibration spectrum of mechanical graphene is characterized by Dirac cones serving as sources of topological nontriviality. We find that the spectrum has dramatic dependence on the spring tension at equilibrium as a natural control parameter, i.e., creation and annihilation of the Dirac particles are realized as the tension increases. Just by rotating the system, the manipulated Dirac particles lead to topological transition, i.e., a jump of the “Chern number” occurs associated with flipping of propagating direction of chiral edge modes. This is a bulk-edge correspondence governed by the Newton’s law. A simple observation that in-gap edge modes exist only at the fixed boundary, but not at the free one, is attributed to the symmetry protection of topological phases.

Graphene^1^, a honeycomb array of carbon atoms, has been one of the hottest topics in condensed matter physics in this decade. What is prominent in graphene is existence of massless Dirac fermions, or Dirac cones at the Fermi energy. In general, Dirac cones allow a lot of phenomena distinct from usual metals or semiconductors. An interesting subject is to manipulate Dirac cones^2^. For instance, shifting the Dirac cones in momentum space induces a gauge field^3^. Or, by inducing a mass of Dirac cones (gap opening), topologically nontrivial phases possibly arise^4^,^5^. One direction of recent developments in Dirac and topological systems is exporting the concept beyond conventional solids. Typical example is a cold atom system in which experimental manipulation of Dirac cones is reported^6^. The other example is a photonic crystal governed by the classical Maxwell equation, where Dirac and topological physics have been demonstrated^7^,^8^,^9^,^10^,^11^,^12^. Very recently, mechanical systems obeying the classical Newton’s equation of motion are also discussed in the context of Dirac cones or topological edge states^13^,^14^,^15^,^16^,^17^,^18^,^19^,^20^,^21^. It is also worth noting that systems in which electromagnetic waves and mechanical vibrations, or photons and phonons, are coupled to each other can also be interesting playground for the topological physics^22^,^23^. A great advantage of these kinds of artificial systems is their controllability, which enables us to realize phenomena that are difficult in solids, such as merging of Dirac cones^24^.

In this paper, a honeycomb spring-mass model^25^, dubbed as mechanical graphene, is investigated as a typical mechanical system having Dirac cones in the frequency spectrum. We propose a simple and feasible way to manipulate Dirac cones, that is, just applying uniform stretch, or in other words, isotropic negative pressure. More specifically, the uniform stretch is achieved by enlarging the lattice constant with the same springs. (Then, the distance between the nearest neighbor mass points is not necessarily same as the natural length of the spring.) As we will see later, the uniform stretch modifies tension of springs, which controls the relation between the transverse and longitudinal waves, and affects the frequency spectrum. As a result, we observe creation, migration, and annihilation of Dirac cones. When the time reversal symmetry is broken by uniform rotation, the manipulated Dirac cones lead to a topological transition, which is detected as a jump in the “Chern number”^18^,^26^, and a flip in the direction of propagation of “chiral edge modes”^27^,^28^. Not only proposing a practical way to manipulate Dirac cones, we also demonstrate the state-of-art idea of topological phenomena, symmetry protection, is at work in this system by focusing on the boundary condition dependence of edge states.

Mechanical graphene consists of mass points with mass *m* 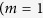 is assumed for simplicity), springs with spring constant 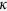 and natural length 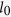, where mass points are aligned in the 2D honeycomb lattice with 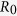 as distance between the nearest mass points. [See Fig. 1a.] Motion of mass points is also restricted in the 2D space, i.e., the out of plane motion is prohibited. As we have noted, we do not restrict ourselves to the situation with 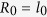. When 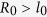, each spring gives finite force even in equilibrium, which means that we should make the system to avoid shrinkage as a whole. For instance, we need to support the system by appropriate choice of the boundary condition. Dynamical variables for this model are deviations of mass points from the equilibrium positions, written as 
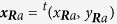
, where ***R*** denotes a lattice point, and *a* is a sublattice index.

## Results

### Formulation

**Figure 1:**
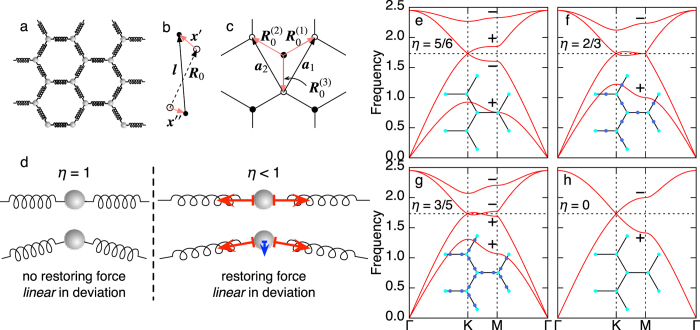
(**a**) Schematic picture of the mechanical graphene. (**b**) Definitions of 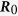, 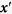, and 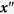. (**c**) The unit vectors 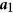 and 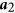, and the vectors connecting the nearest neighbor sites, 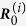 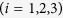. (**d**) Schematic illustration of the role of *η*. (**e**–**h**) Dispersion relations for several *η*. (**e**) 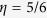. (**f**) 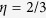. (**g**) 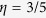. (**h**) 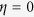. Insets show the positions of the Dirac cones in the Brillouin zone. Cyan and blue dots represent the Dirac cones. + and − signs on the M-point are eigenvalues of the inversion operator.

In mechanical graphene, the elastic energy cost for finite 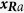 has an special importance. Here, we see it for a pair of mass points, introducing parameters 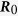, 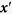, and 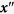 as in Fig. 1b. Throughout this paper, we adopt an assumption that all springs have good linearity, i.e., the energy of a spring is always written as 
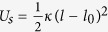
, where *l* is the length of the spring at that moment, 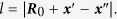 Then, up to second order in 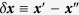, which is required to investigate harmonic oscillation around equilibrium, we have


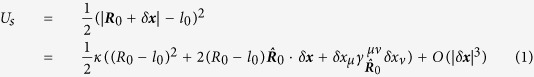


with 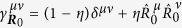, 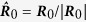, and 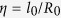. Note that if 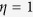, the system is not stretched, while if 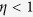, the system is stretched. Summation over indices appearing as a pair is assumed 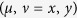. This formula indicates that 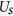 strongly depends on the angle between 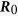 and 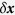 for 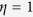, while 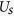 is independent of the direction of 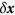 for 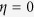. For 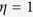, no force is given by a spring if 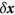 is normal to 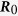, since such motion only leads to the rotation of the spring without elastic energy cost. On the other hand, for 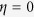, there is finite force even for 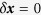, and then the motion with 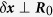 can increase the energy since there is a finite component of force normal to 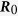. In short, *η* controls the relation between the restoring forces for 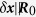 and 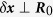, or in other words, the relation between the longitudinal and the transverse wave modes.

The physical meaning of the last statement is illustrated in Fig. 1d. There, we consider the case that a mass point is supported by two linearly arranged springs. For 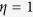, the illustrated sidewise shift does not lead to harmonic oscillation since the potential energy cost for this shift is not quadratic but higher order in the shift size. On the other hand, for 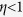, the sidewise shift leads to harmonic oscillation, since the restoring force given as a partial component of the force by the springs is linear in the shift size. So, the motion in perpendicular to the spring direction, which corresponds to a transverse mode, is significantly affected by *η*. In contrast, the motion in parallel with the spring direction, which corresponds to a longitudinal mode, is not affected by *η* as far as good linearity of the springs is assumed, since with good linearity, the elastic energy has a constant second derivative with respect to the spring deformation.

Having this in mind and assuming the periodicity of the system, we introduce new dynamical variables 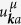 and 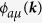 as 
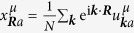
 and 
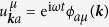
 in order to obtain normal modes. Then the equation to be solved reduces to


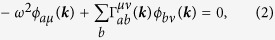


where 
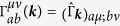
,


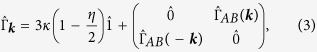


and 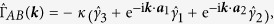 with 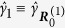, 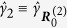, and 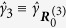. [See Fig. 1c.] Practically, the *ω*-spectrum is obtained by diagonalizing 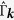. It is worth noting that except the constant diagonal elements, 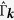 has no term connecting the same kind of sublattices. Since the constant diagonal elements only add a constant contribution to 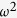, we say that 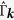 has a “chiral” symmetry, since 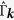 anticommute with 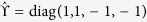 if the constant diagonal part is subtracted. In fermionic systems, the chiral symmetry plays important roles in various situations. For instance, it stabilizes Dirac cones in 2D cases^29^.

Now, the problem is quite similar to the phonon problem in graphene^30^,^31^,^32^,^33^,^34^. However, it is difficult to control *η* in a wide range in real graphene. Furthermore, the effective model for graphene phonon does not respect the “chiral symmetry”, which plays important roles in topological characterization. This is because if the vibration in graphene is modeled by a spring-mass model, it requires springs connecting the next nearest neighbor sites and more.

### Manipulation of Dirac Cones

Now, we follow the *η* dependence of the *ω*-spectrum [Fig. 1e–h]. Throughout this paper, the spring constant is scaled as 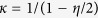 in order to make the total band width constant. For 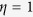 (not shown), the second and third bands of 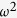-dispersion is same as the one in the nearest neighbor tight-binding model for graphene (NN-graphene) in which Dirac cones are there at K- and K’-points, but the first and the fourth bands are exactly flat and stick to the top and bottom of the other bands. (See Ref. 18.) Note that 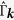 at 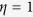 is essentially same as the Hamiltonian of *p*-orbital honeycomb lattice model^35^. The *ω*-dispersion for 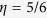 is shown in Fig. 1e. It is similar to the one for 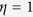 and the Dirac cone is still there at the K- and K’-points, but the first and the fourth bands are no longer flat. At 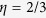 [Fig. 1f], the gap at the M-point between the second and the third bands closes. In the vicinity of the M-point, the dispersion is linear in Γ-M direction but quadratic in M-K direction. Such a linear vs quadratic dispersion is characteristic for merging of a Dirac cone pair^24^. In fact, soon after *η* becomes less than 2/3, two new Dirac cones other than those at the K- and K’- points appear on M-K lines. [See Fig. 1g.] Due to the three fold rotational symmetry, there are six new Dirac cones in the whole Brillouin zone. The new Dirac cones move from the M-point to the K-point as *η* approaches to zero. For small *η* where the new Dirac cones get close to the K- and K’-points, the dispersion relation looks like the one for bilayer graphene with trigonal warping^36^. Finally, at 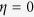 [Fig. 1h], the new Dirac cones are absorbed to the original Dirac cones at the K- and K’-points, and the dispersion becomes like the one of NN-graphene with extra double degeneracy. In 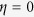 limit, there is no distinction between the longitudinal and transverse waves, and then, oscillations in *x*- and *y*-direction decouple, which leads to the two-fold degeneracy.

The transition at 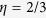 is characterized by parity of the “Herring number”, which is defined for electronic systems as^37^


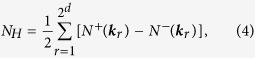


where *d* is spatial dimension, 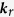
s are the time reversal invariant momenta, and 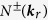 is the number of occupied states with parity eigenvalue ±1 at 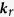. This number is also used to obtain 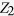 topological number of the inversion symmetric topological insulator, or to check the existence of Dirac cones^38^. For 2D cases, parity of 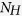 determines the parity of the number of Dirac cones in half of the Brillouin zone as explained below. In Ref. 39, in order to detect Dirac cones in generic 2D systems, we have used the Berry phase defined as


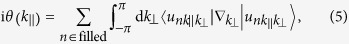


where 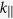 and 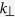 are two momenta in independent directions, and 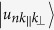 is a Bloch wave function^39^,^40^. When the system respects both of the time reversal and spatial inversion symmetry, 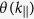 is quantized into 0 or *π* for the spinless case^39^. As far as the quantization is kept, 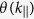 has to show a jump when it changes as a function of 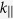, but the jump should be associated with a singularity of the band structure that is nothing more than a Dirac cone. Having this in mind, we can say that if 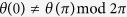 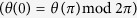, odd (even) number of Dirac cones exist in the region 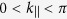, i.e., half of the Brillouin zone. On the other hand, the inversion symmetry gives us a relation^38^,^41^.


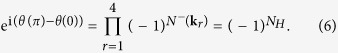


Therefore, the Herring number has an ability to detect the number of Dirac cones. This idea has been applied to the 2D organic Dirac fermion systems^41^,^42^. It is also possible to apply the idea to “mechanical” graphene. We have already seen that the number of Dirac points in the half of the Brillouin zone is odd (one) in 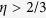 and even (four) in 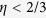. In Fig. 1(e–h), the eigenvalues of the inversion operator


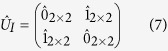


at the M-point are indicated for each band. We notice that at 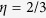, the positive and negative eigenvalues at the M-point are interchanged. Since there is three distinct M-points in the Brillouin zone, this interchange results in the parity change of 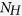. Note that Herring originally considers only 3D cases, and in his paper, 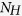 is derived to detects the number of degeneracy loops in 3D Brillouin zone, instead of Dirac cones.

### Edge Modes and Symmetry Protection

**Figure 2:**
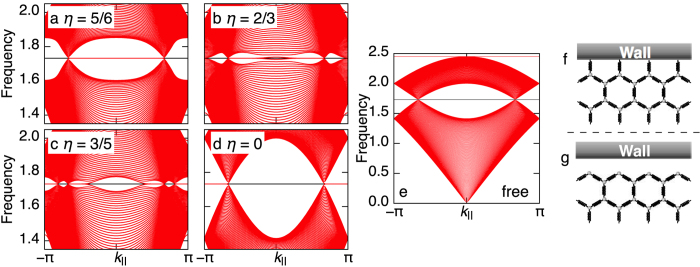
Edge spectra as a function of the momentum along the edge for several *η* = 1 with the fixed boundary condition. (**a**) 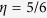. (**b**) 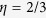. (**c**) 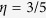. (**d**) 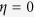. (**e**) Edge spectrum for the free boundary condition with 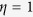. (**f**,**g**) Schematic pictures of the f fixed and (**g**) free boundary conditions.

Next, we see edge states using ribbon geometry with zigzag edges. In this case, the equation to be solved is similar to equation (2), but with larger matrices, since the Fourier transformation is applicable only in one direction. Here, we adapt the fixed boundary condition, that is, the mass points at the boundary are connected to the springs fixed to the wall. [See Fig. 2f,g.] The obtained frequency spectra for several values of 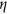 as functions of 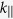, momentum parallel to the edge, are shown in Fig. 2a–d. For 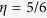, we observe in-gap edge modes near 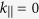 as in the case of 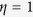
18. The edge modes forms a flat band, since the “chiral symmetry” is preserved at the fixed boundary. At 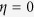, the system exactly inherits the property of NN-graphene, i.e., the edge modes are found near 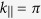 instead of 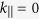. In short, the position of the edge modes in the edge Brillouin zone is switched between near 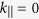 and 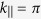 as 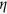 changes. Namely the new Dirac cones emerged from the M-points bring new edge modes near 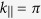 and take away the edge modes near 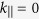.

**Figure 3:**
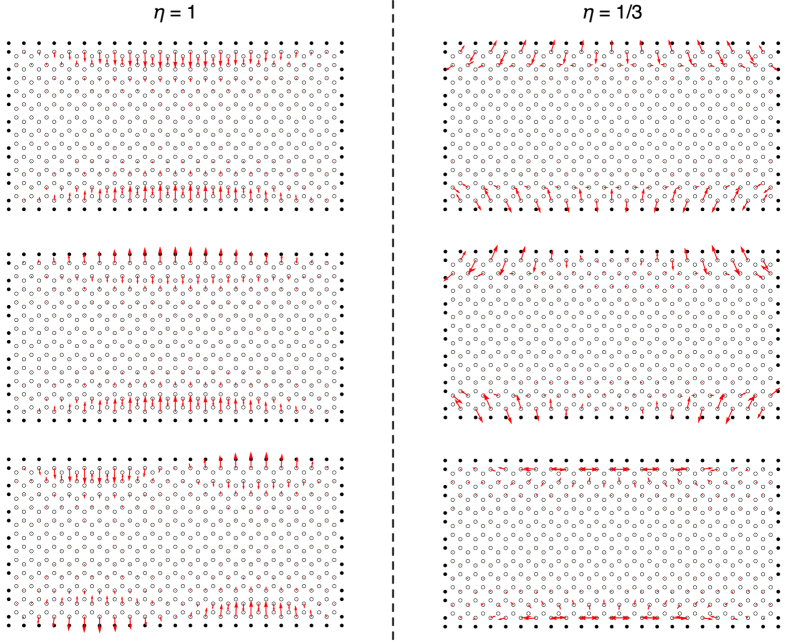
Real space picture of the typical eigenmodes that are localized to the edge for the finite size rectangular system with fixed boundary condition. 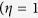
 for the left panels and 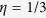 for the right panels.) In order to have the desired shape and boundary condition, we fixed mass points represented as filled dots in the figure. The open dots represent the equilibrium positions for the mobile mass points.

The difference in momenta along the edge is reflected in the oscillation pattern in the real space. In order to convince this expectation we perform a calculation using a rectangular system with long zigzag edges and short armchair edges. In Fig. 3, the typical eigenmodes whose frequency is close to 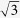, a frequency of the bulk Dirac points, is shown for 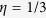 and 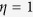. For 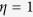, the mass points at the boundary basically oscillate in phase with neighboring mass points on the edge with large wave-length background, reflecting the fact that the edge modes are existing near 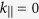 in the momentum space. On the other hand, for 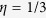, the neighboring mass points on the edge tend to oscillate anti-phase, reflecting the edge mode position in the momentum space. For 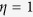, the mass points at the boundary oscillate approximately normal to the boundary, while for 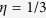, oscillation in parallel to the boundary is also allowed. In Fig. 3, only the typical eigenmodes are shown, but we have confirmed that the above statements are basically valid for the mode near 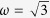. Therefore, the oscillation pattern of the eigenmode localized at the boundary can be used to detect the phase transition associated with the Dirac cone merging experimentally.

In order to see the importance of the boundary condition, the *ω*-spectrum for 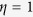 obtained with the free boundary condition, where the springs at the boundary are simply removed, is shown in Fig. 2e. (Note that 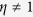 does not go well with the free boundary condition since the finite tension results in the deformation at the boundary.) We find no edge mode in the gap. This difference between the two boundary conditions is deeply related to the symmetry protection of topological phases, which is one of the most important concept in modern theory for topological matters. In the real graphene case, it is possible to assign a topological origin of the edge modes^43^. There, the chiral symmetry plays an important role in defining the bulk topological number, the quantized Berry phase, and preserving in-gap edge states. The fixed boundary respects the “chiral symmetry” of 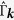. On the other hand, the free boundary does not respects it since the absence of the springs at the boundary leads to *nonuniform* diagonal elements in 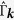. Therefore, even the bulk topological property is same, the topological edge states are not necessarily preserved at the free boundary in contrast to the fixed boundary, which reflects the manifestation of the symmetry protection.

### Time Reversal Symmetry Breaking and Chiral Edge Modes

Lastly, we break the time reversal symmetry by uniform rotation as a whole with angular frequency Ω, which brings two new forces, the centrifugal and the Coriolis force. For simplicity, only the Coriolis force, which is first order in Ω, is taken account of since the centrifugal force, which is second order in Ω, is negligible for small Ω. The equation of motion becomes^18^.


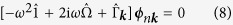


where


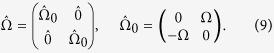


It is no longer possible to reduce the problem to diagonalization of 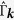. Instead, the *ω*-spectrum is obtained by evaluating 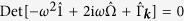 analytically, and solving a quartic equation of 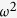. After obtaining *ω*, 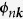 is derived as a nontrivial solution for equation (8). For 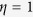, the finite Ω induces a gap at the Dirac point^18^. By solving equation (8), it is confirmed that such a gap opening remains at work also for 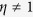. At some critical point 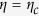, the gap is closed at the M-point. Then, for *η* less than 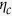, the system is again fully gapped except 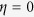 where the gap is again absent. 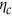 depends on Ω, but it is close to 2/3 in the small Ω limit.

For the topological analysis of this gap closing and opening, we define the Chern number *C* as


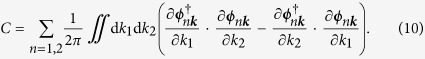


The summation is taken over 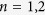, since we are now focusing on the gap between the second and third bands. *C* can be evaluated with a well established numerical method^44^. With 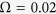, a representative value for small enough Ω, 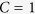 for 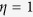, which is consistent with the previous work^18^. As *η* is reduced, the Chern number jumps from 1 to −2 at 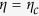, which is close to 2/3 for small Ω. This transition is well understood from the fact that each Dirac cones existing for the time reversal invariant case carries one-half contribution to the Chern number.

In order to have a vivid view on this topological transition, we perform a calculation on a triangular system with zigzag edges. The Chern number for the mechanical system itself is not a physical observable but the corresponding edge states reflecting nontrivial bulk can be observed experimentally^28^. Here, again, the fixed boundary condition is applied, i.e., the system must be fitted in a triangular frame. We consider a forced oscillation problem by picking up one mass point and applying a force that is sinusoidal in time with angular frequency 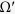, which is explicitly written as 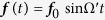. An element of 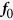 is finite only when it corresponds to the picked mass point. Now, equation to be solved is rewritten as


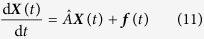


where 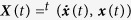,


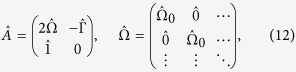


and 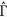 is a dynamical matrix. Assuming that the system is at rest at 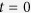, a formal solution of equation (11) is


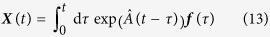


which is explicitly evaluated by diagonalizing 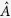 and perform the integration in equation (13).

**Figure 4:**
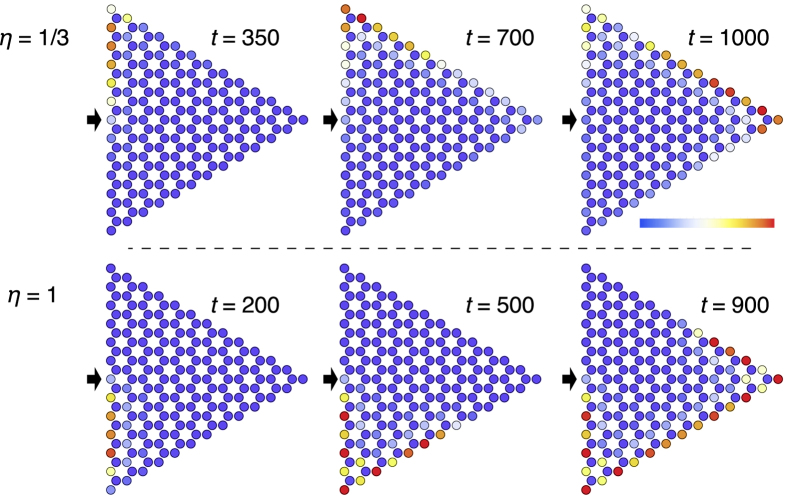
Snapshots of the time evolution of the system with Ω = 0.05. The color map indicates the kinetic energy of each mass point averaged over the time range 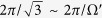. The external force is applied to the mass points indicated by thick arrows. Upper panels: 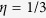 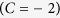. Lower panels: 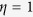 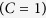.

The selected snapshots of the time evolution for 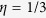 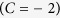 and 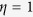 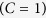 are shown in Fig. 4. Here, the direction of force is chosen to be normal to the edge. We use 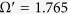 for 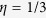 and 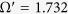 for 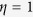 in order to excite edge modes efficiently. Owing to these choices, the oscillation amplitude is localized near the edge. Following the time evolution of the oscillation, we notice that the oscillation amplitude propagates in the fixed direction, which indicates existence of the chiral edge modes. When the “wave front” of the oscillation reaches to the corner, it goes to the next edge instead of being reflected. For 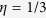 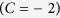, the wave front propagates clockwisely, while for 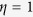 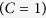, it propagates counterclockwisely. Interestingly, the difference between 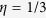 and 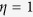 resides in the relation of the transverse and longitudinal modes, and the symmetry of the system is kept intact. Nevertheless, it reverses the direction of the motion of the chiral edge modes.

Before closing, we make a comment on the effects of dissipation. Assuming that the frictional force is proportional to the velocity, the effects of friction are easily incorporated by slightly modifying 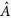. Then, although the friction prevents the long distance propagation of the chiral edge modes, we can still observe the unidirectional nature of the chiral edge modes as far as the friction is enough small. Very recently, an interesting aspect of Dirac cones in photonic system with radiational leakage is reported^45^. Then, addressing the relation between radiational loss in photonic systems and frictional dissipation in mechanical systems is an interesting future subject.

## Discussion

To summarize, we first propose a simple and feasible way, uniform stretch of the system, to control the vibration spectrum of mechanical graphene. An interesting point of the proposed procedure is it does not involve symmetry breaking, but we observe generation, migration, and creation of Dirac cones. It is also shown that the transitions associated with the motion of Dirac cones are detected by observing real space pattern of the edge modes. For the case without the time reversal symmetry, an intimate relation between the bulk topological number and chiral edge modes in a mechanical system is established, which indicates universality of the bulk-edge correspondence, which is one of the most important concepts in the topological approach. In addition, the boundary condition dependence of the edge modes is explained in terms of the symmetry protection of the topological state, which reveals new aspect of the symmetry in classical mechanical systems.

## Additional Information

**How to cite this article**: Kariyado, T. and Hatsugai, Y. Manipulation of Dirac Cones in Mechanical Graphene. *Sci. Rep.* **5**, 18107; doi: 10.1038/srep18107 (2015).
